# Microbial Relevant Fouling in Membrane Bioreactors: Influencing Factors, Characterization, and Fouling Control

**DOI:** 10.3390/membranes2030565

**Published:** 2012-08-15

**Authors:** Bing Wu, Anthony G. Fane

**Affiliations:** 1 Singapore Membrane Technology Centre, Nanyang Technological University, 1 Cleantech Loop, CleanTech One #06-08, Singapore 637141, Singapore; Email: agfane@ntu.edu.sg; 2 Department of Civil and Environmental Engineering, Nanyang Technological University, Singapore 639798, Singapore

**Keywords:** extracellular polymeric substances, membrane fouling, microbial community structure, microbial flocs, microbial soluble substances

## Abstract

Microorganisms in membrane bioreactors (MBRs) play important roles on degradation of organic/inorganic substances in wastewaters, while microbial deposition/growth and microbial product accumulation on membranes potentially induce membrane fouling. Generally, there is a need to characterize membrane foulants and to determine their relations to the evolution of membrane fouling in order to identify a suitable fouling control approach in MBRs. This review summarized the factors in MBRs that influence microbial behaviors (community compositions, physical properties, and microbial products). The state-of-the-art techniques to characterize biofoulants in MBRs were reported. The strategies for controlling microbial relevant fouling were discussed and the future studies on membrane fouling mechanisms in MBRs were proposed.

## 1. Introduction

A membrane bioreactor (MBR) consists of a bioreactor and a membrane separator (located inside or outside of the bioreactor) which replaces the secondary clarifier in the conventional activated sludge process. In MBRs, the key role of bacteria is to decompose organic/inorganic matters in the influent; the membrane separates the microorganisms and some macro-molecules, and allows the water and dissolved species to pass through. MBRs offer several benefits compared to the conventional activated sludge process, such as superior effluent quality, small footprint, and reduced waste sludge production. The application areas of MBRs have been increasing sharply with process innovations and significant cost reductions of membrane modules over the years [1,2]. 

**Figure 1:**
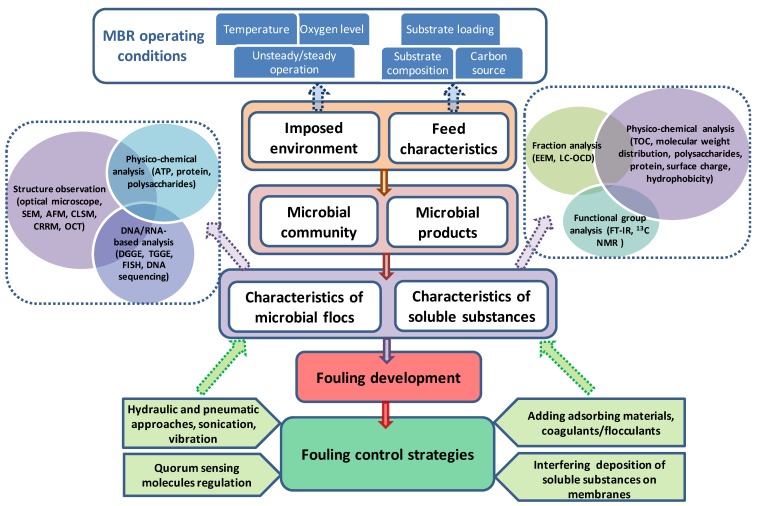
Schematic illustration showing the contents of this review.

There are still a few technical challenges of MBRs, such as limited knowledge on membrane lifespan and membrane integrity, environmental concerns on the wastes of chemicals for membrane cleaning. Another major drawback of MBR technology is membrane fouling, which reduces productivity and increases operating and energy costs of MBRs [3,4]. Fouling in MBRs is primarily caused by microbial deposition/growth and microbial product accumulation on membranes. Characteristics of microorganisms and microbial products in MBRs strongly depend on operating conditions of MBRs. Therefore, this review summarized the factors in MBRs that influence microbial behaviors (community compositions, physical properties, and microbial products) and affect the interactions of microbes/microbial products with membranes. To select a suitable and effective fouling control method, the dominant foulants in MBRs need to be characterized. Accordingly, the recently developed techniques on biofoulant identification were updated in this review. Finally, based on various membrane fouling mechanisms, membrane fouling control strategies were suggested in this review (Figure 1).

### 1.1. MBRs Application and Development

MBRs have wide applications in treatment of industrial and municipal wastewater as well as landfill leachate. Another attractive application area of MBRs is pretreatment of reverse osmosis (RO) and nanofiltration (NF) process [5,6]. Recently, alternative MBR configurations and enhanced hybrid MBRs have received more attention due to their excellence in treatment efficiency improvement, membrane fouling alleviation, and energy saving. For example, MBRs were either coupled with other units such as upflow anaerobic sludge blanket (UASB) and aerobic/anoxic process or operated with aerobic granular sludge to achieve simultaneous removal of carbon, nitrogen, and phosphorus in the wastewaters [7,8]. Using thermally-driven membrane distillation process instead of pressure-driven membrane filtration process, Phattaranawik and Fane proposed a novel membrane distillation bioreactor to achieve excellent water quality comparable to RO permeate quality [9,10]. MBRs utilizing forward osmosis (FO) membranes have been reported currently considering lower fouling potential and less energy consumption compared to the conventional MBR-RO/NF process [11,12,13,14]. In recent years, many efforts have been made on the development of the anaerobic MBRs, which favor higher-strength wastewaters (e.g., food wastewaters, swine manure, landfill leachate, *etc.*) and also can efficiently treat municipal wastewater. Meanwhile, anaerobic MBRs provide the production of energy from the generated biogas [15,16,17]. Microbial fuel cell (MFC) is now actively pursued as a promising technology to generate electric power using wastewaters. Wang *et al.* combined a MBR with a MFC to develop an innovative bioelectrochemical membrane reactor system to simultaneously treat wastewaters and recover energy [18]. Zhang *et al.* further proposed to integrate FO membranes into an MFC for water reuse and seawater desalination. It was found that the FO-MFC (2.39 W/m^3^) generated more electricity than the MFC (2.07 W/m^3^) when using seawater as catholyte [19].

With stricter discharge standards and increased requirements for water re-use, the future challenges for MBRs will focus on scale-up, ease of operation, simplified membrane cleaning and replacement strategies, peak flow management, and energy efficiency. On the other hand, the capital cost of the MBRs is mainly associated with the cost of the membrane modules. Thus, investigations on membrane lifespan and innovation in membrane materials with minimum fouling and cost are crucial for the further development of MBRs [4,20]. Many studies have indicated that coating functional molecules on the membrane surfaces can effectively alleviate fouling [21,22]. Moreover, preparation of composite membranes with nanoparticles is of high interest in recent research studies. Addition of low quantity of nanoparticles such as nano-sized silica, carbon nanotubes, alumina, silver, zirconia, gold, palladium, titanium dioxide, boehmite, or zinc dioxide, *etc.* into membrane structures could enhance membrane performances by decreasing hydrophobicity and improving porosity, selectivity, conductivity, or roughness of membranes [23,24,25]. Recently, there have been increasing concerns on biomimetic behaviors of natural cellular membranes. Researchers have successfully applied this concept in fabricating membranes by incorporating water channel proteins (e.g., aquaporin) into membrane matrixes for improving membrane permeability [26,27,28,29]. Clearly, the innovated membrane materials provide better performances. However, to date, almost no real application of these novel membranes in pilot plant MBRs has been described in the literature. Future research should be considered with a focus on feasibility, stability, and life-cycle assessment of the novel membranes in real MBRs.

### 1.2. Membrane Fouling and Potential Foulants

Membrane fouling is attributed to the larger soluble molecules plugging and narrowing the pores of membranes, or the particles/colloids depositing on the membrane surfaces to form a cake layer [30,31]. In MBRs, microbial flocs, individual microbial cells, microbial metabolic products, and non-degraded matters from wastewaters are considered as potential foulants. In the initial stage (*i.e.*, interaction of membranes with potential foulants), the substances having sizes less than/comparable to membrane pores easily block the membrane pores; simultaneously, the greater-size substances tend to form cake layers on membranes. On a long-term basis, the formed cake layers unable to be removed by physical cleaning perform as a secondary membrane. The foulants may fill in the voids of the formed cake layers or contribute to increase cake layers.

Membrane fouling phenomena and mechanisms in MBRs are the most heavily investigated areas in the MBR publications. Of particular interest are extracellular polymeric substances (EPS) in MBRs, which are excreted or autolyzed by microorganisms and are believed to be a major contributor to membrane fouling. EPS consist of various organic substances, including polysaccharides, proteins, lipids, and also a component of nucleic acids and other bio-polymers. EPS are not only present dissolved or suspended in the solution (soluble EPS), but also attached on the surface of bacteria in flocs (bound EPS). Generally, soluble EPS are obtained by removing the microbial flocs from the mixed liquor using centrifugation or filtration methods. Bound EPS from the cell surfaces is required to be extracted. Previously reported exaction methods include physical extraction methods (centrifugation, ultrasonication, and heating), chemical extractions (ammonium hydroxide, sodium hydroxide (NaOH), ethylenediamine tetraacetic acid (EDTA), sulfuric acid, trichloroacetic acid, boiling benzene, Tris/HCl buffer, phosphate buffer/heat, formaldehyde/NaOH), and cation exchange resin. The compositions of bound EPS as analyzed largely depend upon the methods used for extraction [32,33,34].

Based on the distribution of EPS, the mixed liquors of MBRs are considered to contain two fractions: Microbial flocs and supernatant including colloids and solutes. Recent studies have attempted to quantify the fouling caused by each fraction of the mixed liquor, although the results are inconsistent. Chang and Lee observed that the resistance of the cake layers formed by microbial flocs appeared to determine the overall resistance at various SRTs [35]. Defrance *et al.* concluded that flocs and particulates predominantly contributed to membrane fouling [31]. Lee *et al.* found that the relative contributions of supernatant containing colloids and solutes in the mixed liquor to membrane fouling were 37%, 28% and 29% at SRTs of 20, 40 and 60 days respectively, which reveals microbial flocs are major foulants [36]. Bae and Tak also claimed that cake layer formation caused by microbial flocs constituted the main fouling mechanism [30]. 

However, other researchers have identified the soluble compounds or colloids as prime important factors in causing membrane fouling in MBRs [37,38,39,40]. In these studies, various terms have been used to define these soluble/colloidal substances in MBRs based on different research concerns, such as soluble EPS [41], soluble microbial products (SMP) [42,43,44], biopolymers or biopolymeric clusters (BPC) [45,46], biomacromolecules [47], dissolved organic matter (DOM) [48,49], and transparent exopolymer particles (TEP) [50,51,52,53,54], which follow different sample preparation procedures (centrifugation or filtration) and analytical protocols. Recently, the TEP in MBRs have been paid more attention. TEP was initially investigated in marine ecology [55] as the excreted substances of bacterial cells. The small and sticky nature of TEP provides them a possibility to adhere to a surface, and mobilize bacteria to form biofilm matrix. Meanwhile, TEP contain organic substances and therefore provide nutrients for bacterial growth and development of biofilm [50,51,52,53,54]. Whether TEP can be considered as a fouling indicator in MBRs and how the relationship is between EPS and TEP are still under question. Although there are various definitions on soluble microbial relevant substances, these molecules are believed to induce membrane fouling by causing membrane pore clogging and narrowing, or forming gel-like cake layers on membranes. 

It is worth mentioning that the contributions of the potential components (*i.e.*, microbial flocs or SMP) to fouling in MBRs are variable in these studies, mainly because of various feeding wastewaters, membrane properties, hydrodynamic conditions, and importantly, physiological characteristics of microbes which were determined by the reactor operating conditions [30,37]. In addition, at the moment, due to lack of standard protocols to identify the dominant foulants in MBRs, it may not be entirely reliable to directly compare the findings in different studies. This area clearly needs more work in future. 

## 2. Key Factors Influencing Microbial Behaviors in MBRs

In MBRs, microorganisms maintain their growth by oxidation and synthesis as well as endogenous respiration processes using organic/inorganic substances in the wastewaters. Meanwhile, metabolic products excreted from living microorganisms and lysis substances from dead cells are generated. Membranes submerged into reactors inevitably interact with these substances under hydrodynamic conditions. Importantly, once first layer was formed on membrane surfaces by microorganisms and their metabolic matters, further adherence of foulants on membrane surfaces will be governed by surface properties and structure natures of the initial cake layer. Therefore, the characteristics of microbial flocs and SMP perform key roles on their interactions with membranes in MBRs.

Generally, microbial growth and metabolism depend on feed characteristics and imposed environment (e.g., oxygen level, temperature, steady-state/unsteady-state operation). Thus, MBR operating conditions involved in these factors influence the microbial behaviors such as the presence of microbial species, physiological characteristics of microbial flocs, and their metabolic products. 

### 2.1. Feed Characteristics

MBRs have been applied to treat a wide range of industrial and municipal wastewater with variable nutrient inputs (e.g., carbon, nitrogen, and phosphorus contents). Substrate loading and composition are found to be the primary factors influencing bacterial community in MBRs. Wu *et al.* illustrated that bacterial community structure dynamically shifted in different ways under various organic, nitrogen, or phosphorus loadings in MBRs [56,57]. Ahmed *et al.* reported that when different external carbon sources were provided in MBRs, dominance of α, β, γ-subclass of *Proteobacteria* was dissimilar [58]. Concomitantly, the differences in the nutrient sources could influence physiological properties of biomass (e.g., concentration, particle size, viscosity, floc structure) as well as chemical compositions and distributions of EPS in MBRs, which have an effect on membrane fouling profiles [56,57,58,59,60,61,62]. Wu *et al.* reported that the membrane fouling tendency of biomass in the low-loading MBR (0.57 g COD/L day) was insignificantly different from that in the medium-loading MBR (1.14 g COD/L day), which was apparently lower than that in the high-loading MBR (2.28 g COD/L day). This is attributed to the higher bound EPS contents in the high-loading MBR. On the other hand, the nutrient amount available for bacteria is inversely related to sludge retention time (SRT) employed in MBRs. For example, at the same organic loading, MBRs with a shorter SRT have a higher food to microorganisms (F/M) ratio. A large body of research pointed out that a high F/M ratio in the MBR is beneficial to bacteria for the synthesis of cellular material (including growth of new cells and production of excreted substances), which as a result aggravates membrane fouling [63,64,65,66]. 

### 2.2. Imposed Environment

#### 2.2.1. Oxygen Level

Aerobic growth of microorganisms is strongly dependent on the amount of oxygen available because oxygen is a key terminal electron acceptor to yield energy in their metabolic pathways. In MBRs, imposed dissolved oxygen (DO) level may facilitate propagation of some microbial species, but may disfavor others. Vibration of oxygen amount in a reasonable range (e.g., high DO *vs.* moderate DO) may not markedly change the microbial community compositions in the MBRs. Almost similar dominant species, for example, *Betaproteobacteria*, *Dechloromonas*, *Rhodocyclus*, *Comanonas*, and *Nitrospira*, are found under such DO conditions. However, lowering DO levels to a threshold (e.g., less than 0.5 mg/L) led to noticeable changes in the microbial community structure (*i.e.*, enhanced denitrifying bacterial growth) and distinct decreases of diversity of predominant microbial populations in MBRs [67,68,69]. On the other hand, the oxygen level available in MBRs influences microbial metabolisms such as generation, composition, and distribution of EPS [56,67]. Accordingly, membrane performances associated with microbial behaviors can be greatly affected by DO levels. Gao *et al.* emphasized that insufficient DO amounts in MBRs facilitated EPS production in the mixed liquor and EPS accumulation in the cake layers, which induced higher membrane fouling rates [67]. In other studies, it was observed that lowing DO levels reduced the sizes of microbial flocs, which tend to form dense and compact cake layers on the membranes and give rise to higher resistances [68,70].

#### 2.2.2. Temperature

In MBRs, microorganisms use their enzymes to hydrolyze and degrade the organic/inorganic matters and the levels of enzyme activities are sensitive to seasonal temperatures. The activities of some enzymes (such as phosphatase and esterase) positively responded to temperature increases in a suitable range, while some enzymes (e.g., glucosidase) may achieve maximum activity at a low temperature when domestic wastewater was treated by the MBR [71,72]. Reduced enzyme activities lead to less biodegradation of organic substances, resulting in higher concentrations of organic substances retained in the reactors. Meanwhile, environmental temperatures influence microbial growth rate and microbial community compositions in MBRs. Favorable temperatures facilitate propagation of suitable microbes, but unsuitable microbial species may disappear or reduce their quantity in the reactors. In some situations, with temperature changing, almost similar microbial community composition may be present in MBRs, but the microbial diversity developed in a highly dynamic pattern [73,74,75,76]. Furthermore, temperatures affect not only properties of microbial flocs such as viscosity and size, but also releasing EPS levels. Miyoshi *et al.* reported that when the temperature decreased from 21.5 to 17.7 °C, almost comparable soluble polysaccharides and protein amounts were observed, while further decreasing the temperature to 12.7 °C significantly induced higher soluble polysaccharides and protein levels in the MBRs [77]. A similar finding was concluded by van den Brink and his colleagues. Therefore, higher membrane fouling rates were obtained at lower temperatures [77,78]. 

#### 2.2.3. Steady-State *vs.* Unsteady-State Operation of MBRs

Stable operation of MBRs is desirable in order to maintain steady reactor performance and membrane filtration process. However, in pilot-plant or full-scale MBRs, unsteady states such as seasonal fluctuation of wastewaters, intermittent feeding, shifts in the oxygen supply, pH change, and discontinuous or irregular disposal of waste sludge may happen. Microorganisms in MBRs respond to these variations by developing suitable microbial community or varying their metabolic and synthesis processes to increase their tolerance. Significant bacterial population changes have been observed in the startup period of MBRs when wastewater compositions, organic loadings, and SRTs were varied, even though the stable MBR performances (such as membrane permeability and organic carbon removal rate) were achieved. [56,61,63]. On the other hand, a few studies pointed out that unsteady organic loading rates led to higher soluble polysaccharides contents in the reactor, which increased fouling rates. Yogalakshmi and Joseph illustrated that the soluble EPS in the MBRs increased by 22%–66% after transient sodium chloride shock. Wu *et al.* observed that when the levels of soluble polysaccharides and soluble TEP in the MBR unexpectedly and suddenly increased due to pH decrease from ~7.0 to ~3.0, the cleaned membranes tended to be more easily fouled compared to the membranes with the initial cake layers formed in a slow TMP increase stage [40,79,80,81]. 

## 3. State-of-the-Art Techniques Characterizing Biofoulants in MBRs

To determine effective membrane fouling control strategies, examination of physical, chemical, and biological properties of these biofoulants on the membranes and identification of major fouling species are key [82]. Recently developed techniques to characterize the biofoulants allow us to understand the natures of fouled membranes. Especially, synergizing the information from various techniques offer us the opportunities to obtain comprehensive knowledge on the foulant development on membranes and provide insights into membrane fouling mechanism of MBRs.

### 3.1. Microbial Flocs

Observation of structure nature of microbial flocs deposited on membranes by optical microscope, conventional scanning electron microscopy (SEM), environmental SEM (ESEM), atomic force microscopy (AFM), confocal laser scanning microscopy (CLSM), confocal resonance Raman microscopy (CRRM), and optical coherence tomography (OCT) is a direct and useful approach to understand fouling behaviors of microorganisms in MBRs. As summarized in recent review papers [82,83,84,85], these visual tools can provide two dimensional (2-D) and 3-D images of fouling layers, can integrate with software to quantify the biovolumes of the foulants, and especially, can identify fouling species and determine their interactions with membranes. 

For example, in ESEM, ruthenium red combined with lysine can enhance the resolution of EPS, which were clearly distinguished from microbial cells in biofilm structures [86]. In AFM, by comparing the roughness of foulants on the membranes, the dominant compositions of foulants (e.g., microbial flocs or SMP) can be determined and the effect of MBR operating conditions on the amounts of foulants on the membranes can be explained [87,88,89]. A recent study further illustrated the interactions between the SMP and membranes by modeling the membrane surface topology based on the statistical parameters originated from AFM. The results indicated that SMP tend to trap in the attractive energy regions as approaching to the membranes, which implies the importance of membrane material and morphology in membrane filtration processes [90]. CLSM can detect different microbial relevant foulants (viable/dead bacterial cells, polysaccharides, protein, lipid, nucleic acids) by staining them with various fluorescence probes and comparing the fluorescence densities [85,91,92]. As a powerful non-destructive tool, CRRM not only can describe the surface morphologies of biofilms, but also can track the microbial community distribution, identify the microbial species, and detect the presence of mineral microparticles. A few studies using CRRM to observe biofilm structure and trace nitrifiers and anammox bacteria using resonance Raman effect of cytochrome *c* have been reported [83,93]. OCT is another fast, high solution, reliable, and non-invasive technique, which has been applied to monitor mesoscale structures of biofilms on membranes [94,95]. 

Physico-chemical analysis can precisely examine the components of attached microbial flocs on the membranes by quantification of microbial cells and bound EPS on the cell surfaces. For microbial cells, total cell count, heterotrophic plate count, and adenosine triphosphate (ATP) are considered as analysis parameters. Compared to traditional cell counting approaches, intracellular and extracellular ATP measurements (indicating viable and dead biomass concentrations respectively) are much faster, more simple and accurate [96]. Importantly, researchers have pointed out that extracellular ATP as a cell-to-cell communication signal can stimulate bacterial adhesion on a surface and facilitate biofilm formation [97]. Therefore, the information of ATP analysis of the foulants can be used to predict membrane biofouling development in MBRs.

Another concern is bound EPS on the cell surfaces, which contribute to form microbial flocs and provide adhesive potential onto membranes to form cake layers. A recently-developed quartz crystal microbalance with dissipation monitoring (QCM-D) technique was successfully applied in studying the adherence and viscoelastic properties of bound EPS of activated sludge from MBRs [91].

DNA/RNA-based analysis can identify microbial community compositions in the deposited microbial flocs on the membranes, which provide more comprehensive, rapid, and concise information on characterization of bacteria from the complex bacterial community. Techniques such as fluorescent *in situ* hybridization (FISH), denaturing gradient gel electrophoresis (DGGE), temperature gradient gel electrophoresis (TGGE), and DNA sequencing offer good opportunities in community analysis, even allowing direct quantification of the presence and abundance of bacterial species. Using these techniques, the microbial communities in the cake layers have been characterized in several published work, especially those associated with cake layer formation, spatial distributions in cake layers, and fouling evolution at different operating conditions of MBRs. In addition, there seems to be markedly difference between the microbial community composition in the mixed liquor and on the membranes [58,63,98,99,100]. Regarding the dominant fouling relevant bacteria, the findings in the literature are slightly different because of various experimental conditions of MBRs. Even in the same reactor, different hydrodynamic conditions may also influence dominant bacteria in the cake layers. Huang *et al.* found that α- and β-Proteobacteria (accounting for 64%–65%) were enriched in the cake layers at a low flux (15 L/m^2^ h), while, α-Proteobacteria and Bacteroidetes were predominant at a high flux (20 L/m^2^ h) [99]. Although many efforts have put on microbial community analysis in MBRs, the challenge is to clarify their fouling associated functionalities. 

### 3.2. Soluble Foulants

In general, soluble foulants are characterized and evaluated by examination of total organic carbon (TOC), molecular weight distribution, polysaccharides, protein, surface charge, hydrophobicity, *etc.* Recently, advanced techniques are actively utilized to characterize soluble foulants. For example, liquid chromatography-organic carbon detection (LC-OCD) has been used to describe the properties of colloidal and soluble organics in MBRs considering molecular size and aromatic characteristics simultaneously [48]. Researchers further combined LC-OCD with UV detector (UVD) and organic nitrogen detector (OND) to estimate the specific UV absorbance at 254 nm and mean oxidation number of carbon and nitrogen in the soluble organic matters [43,101,102]. Using LC-OCD-OND, Huber *et al.* detected five major fractions, namely, biopolymers, humic substances, building blocks, low molecular-weight acids, and low molecular-weight neutrals in the water samples [101]. Filloux *et al.* further identified the proteins in the effluent organic matters responsible for membrane fouling by LC-OCD-UVD-OND [102].

Meanwhile, as a rapid, selective, and sensitive technique, excitation-emission matrix (EEM) fluorescence spectroscopy can obtain fluorescence characteristics of soluble foulants by changing the excitation and emission wavelength simultaneously. Previous studies revealed that several main peaks have been identified from fluorescence spectra of soluble substances in MBRs, such as aromatic proteins (tyrosine and tryptophan, excitation wavelength <250 nm; emission wavelength <350 nm), soluble microbial by-product-like material (excitation wavelength at 250–280 nm; emission wavelength <380 nm), fulvic acid-like substances (excitation wavelength at 200–250 nm; emission wavelength >380 nm); and humic acid-like substances (excitation wavelength >280 nm; emission wavelength >380 nm). By analyzing the distribution of fluorescence regional integration (FRI), the dominant composites of sludge supernatant, soluble foulants, and MBR permeate can be determined, and subsequently the fate and transport of the soluble organic foulants in MBRs can be tracked [43,49]. In a recent MBR study by Meng *et al.*, aromatic protein-like substances and tryptophan protein-like substances were found to be captured by the fouling layer, similarly to the finding in a study by Wang *et al.* [49]. A few other researchers observed that microbial by-product-like substances were predominant in the fouling layers of the MBRs [103,104]. The difference might be related with dissimilar feed wastewaters (raw wastewater *vs.* synthetic wastewater) and various types of MBRs (hybrid MBR *vs.* conventional MBR).

As a powerful spectra analysis tool, Fourier transform infrared (FTIR) spectroscopy can further identify functional groups of organic molecules adsorbed on membrane surfaces in MBRs [105]. Meanwhile, by comparing the peak intensity and peak shift in the FTIR spectra between the virgin and fouled membranes, the dominant foulants can be easily detected. Several typical peaks have been reported to indicate protein and polysaccharides in the soluble foulants. Namely, the peaks at 1640 cm^−1^ (stretching vibration of C=O in amide I), 1540 cm^−1^ (N–H formation in amide II), and at 1240 cm^−1^ (C–N vibration in amide III) reveal the presence of protein [106]. The peaks at 1000–1200 cm^−1^ (C=O bond stretching) are associated with polysaccharides and polysaccharide-like substances [107].

Similarly, an attractive dimension of ^13^C nuclear magnetic resonance (NMR) is to detect protein (peptide peaks at ~160 to ~190 ppm), polysaccharides (secondary alcohol peaks at ~60 to ~90 ppm; glycosidic carbon peaks at ~95 to ~106 ppm), and lipids (peaks at ~0 to ~40 ppm) in the soluble foulants [108]. Meng *et al.* combined ^13^C NMR and EEM to analyze the size-fractionated biomacromolecules in the membrane foulants and found that the macromolecules with different sizes had various abundances and structures of organic compounds. The macromolecules with a size ranging from 100 k Dalton to 0.45 μm had relatively higher polysaccharides contents and mainly contributed to fouling layers [47].

## 4. Effective Strategies Controlling Microbial Relevant Fouling in MBRs

### 4.1. Microbial Flocs-Dominant Fouling Control

Control approaches of membrane fouling cover a large of field of study. Physical cleaning are generally strategies for removing microbial flocs from membrane surfaces, including hydraulic approaches (relaxation, forward flushing, backwashing, backpulsing), pneumatic approaches (air sparging, air lifting, air scouring, and air bubbling), sonication (ultrasound), and vibration [109,110]. A recent study presented a novel MBR fouling control strategy by magnetically induced vibration. The results indicated that magnetically vibration at an intermittent operation mode (2 min on, 2 min off) could ensure a high membrane filtration flux (14–26 L/m^2^ h) at a lower membrane fouling rate than that in the conventional aerated MBRs. Meanwhile, remarkably less energy was required for magnetically vibration-MBRs at the optimal conditions (2.03 kWh/m^3^ *vs.* 6.06 kWh/m^3^ of conventional MBRs) [111].

Biological control of biofilm growth and attachment on membrane surfaces using chemical molecules is a promising alternative for fouling alleviation [112]. Recent studies have demonstrated that extracellular small-molecules signaling involved in cell-cell communication (quorum sensing) play critical roles in biofilm formation in water environments [113,114,115]. For example, quorum sensing molecules (*N*-acyl homoserine lactone, AHL) was present in the MBRs and their activities were correlated with membrane biofouling by regulating bacterial EPS production. Therefore, addition of acylase into the MBRs to inactivate AHL was suggested as a promising anti-fouling method [116]. Xu and Liu proposed to use 2,4-dinitrophenol (DNP) to reduce membrane biofouling. The authors found that DNP could disrupt energy metabolism (*i.e.*, inhibiting ATP synthesis) of microorganisms and enhance biofilm detachment from the membranes [117]. 

### 4.2. SMP-Dominant Fouling Control

As the roles of SMP in membrane fouling have been thoroughly identified, the most important and effective fouling control approach is to minimize their concentrations in MBRs. Careful optimization of operating conditions (SRT and oxygen level) and avoidance of unsteady-state operation should be taken into account.

Lessening interactions between soluble matters and membranes can reduce irreversible fouling and gel-like cake layer formation on membranes. Normally, addition of adsorbing materials (e.g., activated carbon, zeolite) or coagulants/flocculants (e.g., polyamide, polyaluminum chloride, diatomite) into MBRs to absorb or co-precipitate with soluble substances could be effective strategies to control membrane fouling caused by SMP. Addition of diatomite (50 mg/L) into the MBRs could improve membrane performance (fouling rate decreased from 0.47 to 0.11 kPa/day) by reducing the fine colloids and DOM in the reactors. Wu *et al.* compared the effect of powdered activated carbon, zeolite, and coagulants (polyamide, polyaluminum chloride) on membrane filtration treating anaerobic effluent and determined polyaluminum chloride (10 mg/L) as the most effective additive in reducing fouling [118,119]. Johir *et al.* found that granule activated carbon could efficiently remove a range of organic matters including amino acids, biopolymers, humic acid-like substances, and fulvic acid-like substances from the MBR supernatant by adsorption mechanisms, therefore reducing the total membrane resistance from 51 × 10^11^ to 20 × 10^11^ m^−1^ [120]. Koseoglu *et al.* further compared the effects of several strongly cationic charged additives on SMP removal and provided evidences on efficiently reducing SMP up to 72% by a commercial cationic biopolymer [121]. However, more in-depth knowledge is needed on the effect of additives on the long-term operation of MBRs. Additionally, interfering deposition of soluble microbial products onto the membranes has been emphasized as a fouling mitigation method in a few studies. Initially-formed and loose-structured cake layers on membranes can act as a secondary dynamic membranes or prefilters to entrap soluble substances, which facilitate enhancement of membrane long-term performances [80,122]. Teychene *et al.* attempted to use the nano-sized inert particles (polystyrene latex, melamine) to change the morphology of the cake layers formed by the supernatant foulants (*i.e.*, from less porous compressible cake layers to more porous non-compressible cake layers), leading to better membrane filterability as a result [123]. Sun *et al.* noticed that ozonation of the BPC solution (an ozone dose of 0.3 mg/mg TOC of BPC) greatly reduced the BPC size (from 38 to 12 µm) because the ozone also easily attacks glycosidic bonds of the long-chain polysaccharides, resulting in their breakdown into low molecule weight polysaccharides. Meanwhile, this dose of ozone resulted in a decrease of the viscosity of BPC solution (from ~1.2 to ~1.1 mPa s). Reduction of BPC size and viscosity could lower the detrimental role of BPC in membrane fouling [46]. Besides these fouling control strategies, proper selection of the membrane materials also leads to a stable and better MBR performance.

## 5. Conclusions

Since the concept of MBRs was commercialized by the Dorr-Oliver company in the 1960s [124], membrane fouling mechanisms and control strategies have been investigated over half a century. The major concerns on fouling mitigation in MBRs have been shifted from optimization of operating conditions to regulations of microbial behaviors. Especially, a combination of emerging advanced analytical tools and developing molecular microbiology allows us to better understand the characteristics of microbial relevant foulants. Identification of dominant foulants by linking foulant characteristics with fouling development can facilitate determination of effectively fouling control strategies. Although many efforts on fouling control in MBRs have been achieved, consistent and detailed solutions in real cases as well as economic feasibility are still not clear due to complexity of MBRs. Future studies on the functionalities of dominant fouling related microbial species, dynamic behaviors of fouling relevant fractions, feasibility of biological triggers for fouling control on existing MBR plants are suggested.

## References

[1] Judd S. (2008). The status of membrane bioreactor technology. Trends Biotechnol..

[2] Le-Clech P. (2010). Membrane bioreactors and their uses in wastewater treatments. Appl. Microbiol. Biotechnol..

[3] Le-Clech P., Chen V., Fane A.G. (2006). Fouling in membrane bioreactors used in wastewater treatment. J. Membr. Sci..

[4] Meng F., Chae S.R., Drews A., Kraume M., Shin H.S., Yang F. (2009). Recent advances in membrane bioreactors (MBRs): Membrane fouling and membrane material. Water Res..

[5] Alturki A.A., Tadkaew N., McDonald J.A., Khan S.J., Price W.E., Nghiem L. (2010). Combining MBR and NF/RO membrane filtration for the removal of trace organics in indirect potable water reuse applications. J. Membr. Sci..

[6] Kent F.C., Farahbakhsh K., Mahendran B., Jaklewicz M., Liss S.N., Zhou H. (2011). Water reclamation using reverse osmosis: Analysis of fouling propagation given tertiary membrane filtration and MBR pretreatments. J. Membr. Sci..

[7] An Y., Yang F., Chua H.C., Wong F.S., Wu B. (2008). The integration of methanogenesis with shortcut nitrification and denitrification in a combined UASB with MBR. Bioresour. Technol..

[8] Li X., Gao F., Hua Z., Du G., Chen J. (2005). Treatment of synthetic wastewater by a novel MBR with granular sludge developed for controlling membrane fouling. Sep. Purif. Technol..

[9] Phattaranawik J., Fane A.G., Pasquier A.C.S., Wu B. (2008). A novel membrane bioreactor based on membrane distillation. Desalination.

[10] Phattaranawik J., Fane A.G., Pasquier A.C.S., Wu B., Wong F.S. (2009). Experimental study and design of submerged membrane distillation bioreactor. Chem. Eng. Technol..

[11] Achilli A., Cath T.Y., Marchand E.A., Childress A.E. (2009). The forward osmosis membrane bioreactor: a low fouling alternative to MBR processes. Desalination.

[12] Cornelissen E.R., Harmsen D., de Korte K.F., Ruiken C.J., Qin J.-J., Oo H., Wessels L.P. (2009). Membrane fouling and process performance of forward osmosis membranes on activated sludge. J. Membr. Sci..

[13] Lay W.C.L., Zhang J., Tang C.Y., Wang R., Liu Y., Fane A.G. (2012). Factors affecting flux performance of forward osmosis systems. J. Membr. Sci..

[14] Zhang J., Lay W.C.L., Chou S., Tang C.Y., Wang R., Fane A.G. (2012). Membrane biofouling and scaling in forward osmosis membrane bioreactor. J. Membr. Sci..

[15] He Y., Xu P., Li C., Zhang B. (2005). High-concentration food wastewater treatment by an anaerobic membrane bioreactor. Water Res..

[16] Lin H.J., Xie K., Mahendran B., Bagley D.M., Leung K.T., Liss S.N., Liao B.Q. (2009). Sludge properties and their effects on membrane fouling in submerged anaerobic membrane bioreactors (SAnMBRs). Water Res..

[17] Padmasiri S.I., Zhang J., Fitch M., Norddahl B., Morgenroth E., Raskin L. (2007). Methanogenic population dynamics and performance of an anaerobic membrane bioreactor (AnMBR) treating swine manure under high shear conditions. Water Res..

[18] Wang Y.K., Sheng G.P., Li W.W., Huang Y.X., Yu Y.Y., Zeng R.J., Yu H.Q. (2011). Development of a novel bioelectrochemical membrane reactor for wastewater treatment. Environ. Sci. Technol..

[19] Zhang F., Brastad K.S., He Z. (2011). Integrating forward osmosis into microbial fuel cells for wastewater treatment, water extraction and bioelectricity generation. Environ. Sci. Technol..

[20] Shannon M.A., Bohn P.W., Elimelech M., Georgiadis J.G., Marinas B.J., Mayes A.M. (2008). Science and technology for water purification in the coming decades. Nature.

[21] Nady N., Franssen M.C.R., Zuihof H., Eldin M.S.M., Boom R., Schroen K. (2011). Modification methods for poly(arylsulfone) membranes: A mini-review focusing on surface modification. Desalination.

[22] Yu Q., Zhang Y., Wang H., Brash J., Chen H. (2011). Anti-fouling bioactive surfaces. Acta Biomater..

[23] Balta S., Sotto A., Luis P., Benea L., Van der Bruggen B., Kim J. (2012). A new outlook on membrane enhancement with nanoparticles: The alternative of ZnO. J. Membr. Sci..

[24] Kim J., Van der Bruggen B. (2010). The use of nanoparticles in polymeric and ceramic membrane strucutres: Review of manufacturing procedures and performance improvement for water treatment. Environ. Pollut..

[25] Vatanpour V., Madaeni S.S., Rajabi L., Zinadini S., Derakhshan A.A. (2012). Boehmite nanoparticles as a new nanofilter for preparation of antifouling mixed matrix membranes. J. Membr. Sci..

[26] Li X., Wang R., Tang C., Vararattanavech A., Zhao Y., Torres J., Fane T. (2012). Preparation of supported lipid membranes for aquaporin Z incorporation. Colloids Surf. B Biointerfaces.

[27] Duong P.H.H., Chung T.S., Jeyaseelan K., Armugam A., Chen Z., Yang J., Hong M. (2012). Planar biomimetic aquaporin-incorporated triblock copolymer membranes on porous alumina supports for nanofiltration. J. Membr. Sci..

[28] Zhong P.S., Chung T.S., Jeyaseelan K., Armugam A. (2012). Aquaporin-embeded biomimetic membranes for nanofiltration. J. Membr. Sci..

[29] Wang H., Chung T.S., Tong Y.W., Meier W., Chen Z., Hong M., Jeyaseelan K., Armugam A. (2011). Preparation and characterization of pore-suspending biomimetic membranes embedded with Aquaporin Z on carboxylated polyethylene glycolpolymer cushion. Soft Matter..

[30] Bae T.H., Tak T.M. (2005). Interpretation of fouling characteristics of ultrafiltration membranes during the filtration of membrane bioreactror mixed liquor. J. Membr. Sci..

[31] Defrance L., Jaffrin M.Y., Gupta B., Paullier P., Geaugery V. (2000). Contribution of various constituents of activated sludge to membrane bioreactor fouling. Bioresour. Technol..

[32] Domínguez L., Rodríguez M., Prats D. (2010). Effect of different extraction methods on bound EPS from MBR sludges. Part I: Influence of extraction methods over three-dimensional EEM fluorescence spectroscopy fingerprint. Desalination.

[33] Comte S., Guibaud G., Baudu M. (2006). Relation between extraction protocols for activated sludge extracellular polymeric substances (MBR) and MBR complexation properties. Part I: Comparison of the efficiency of eight MBR extractions methods. Enzyme Microb. Technol..

[34] Liu H., Fang H. (2002). Extraction of extracellular polymeric substances (MBR) of sludges. J. Biotechnol..

[35] Chang I.S., Lee C.H. (1998). Membrane filtration characterisitcs in membrane coupled activated sludge—The effect of physiological states of activated sludge on membrane fouling. Desalination.

[36] Lee W., Kang S., Shin H. (2003). Sludge characteristics and their contribution to microfiltration in submerged membrane bioreactor. J. Membr. Sci..

[37] Bouhabila E.H., Aim R.B., Buisson H. (2001). Fouling characterisation in membrane bioreactors. Sep. Purif. Technol..

[38] Chang I.S., Kim S.N. (2005). Water treatment using membrane filtration—Effect of biosolids concentration on cake resistance. Process Biochem..

[39] Wisniewski C., Grasmick A. (1998). Floc Size Distribution in a Membrane Bioreactor and Consequences for Membrane Fouling. Colloids Surf..

[40] Zhang J., Zhou J., Liu Y., Fane A.G. (2010). A comparison of membrane fouling under constant and variable organic loadings in submerge membrane bioreactors. Water Res..

[41] Wang Z., Wu Z., Tang S. (2009). Extracellular polymeric substances (EPS) properties and their effects on membrane fouling in a submerged membrane bioreactor. Water Res..

[42] Jarusutthirak C., Amy G. (2006). Role of soluble microbial products (SMP) in membrane fouling and flux decline. Environ. Sci. Technol..

[43] Jiang T., Kennedy M.D., De Schepper V., Nam S.N., Nopens I., Vanrolleghem P.A., Amy G. (2010). Characterization of soluble microbial products and their fouling impacts in membrane bioreactors. Environ. Sci. Technol..

[44] Ni B.J., Rittmann B.E., Yu H.Q. (2011). Soluble microbial products and their implications in mixed culture biotechnology. Trends Biotechnol..

[45] Wang X.M., Li X.Y. (2008). Accumulation of biopolymer clusters in a submerged membrane bioreactor and its effect on membrane fouling. Water Res..

[46] Sun F.Y., Wang X.M., Li X.Y. (2011). Effect of biopolymer clusters on the fouling property of sludge from a membrane bioreactor (MBR) and its control by ozonation. Process Biochem..

[47] Meng F., Zhou Z., Ni B.J., Zheng X., Huang G., Jia X., Li S., Xiong Y., Kraume M. (2011). Characterization of the size-fractionated biomacromolecules: Tracking their role and fate in a membrane bioreactor. Water Res..

[48] Meng F., Drews A., Mehrez R., Iversen V., Ernst M., Yang F., Jekel M., Kraume M. (2009). Occurrence, source, and fate of dissolved organic matter (DOM) in a pilot-scale membrane bioreactor. Environ. Sci. Technol..

[49] Wang Z., Wu Z., Tang S. (2009). Characterization of dissolved organic matter in a submerged membrane bioreactor by using three-dimensional excitation and emission matrix fluorescence spectroscopy. Water Res..

[50] Villacorte L.O., Kennedy M.D., Amy G.L., Schippers J.C. (2009). The fate of Transparent Exopolymer Particles (TEP) in integrated membrane systems: Removal through pre-treatment processes and deposition on reverse osmosis membranes. Water Res..

[51] de la Torre T., Lesjean B., Drews A., Kraume M. (2008). Monitoring of transparent exopolymer particles (TEP) in a membrane bioreactor (MBR) and correlation with other fouling indicators. Water Sci. Technol..

[52] Bar-Zeev E., Berman-Frank I., Liberman B., Rahav E., Passow U., Berman T. (2009). Transparent exopolymer particles: Potential agents for organic fouling and biofilm formation in desalination and water treatment plants. Desalin. Water Treat..

[53] Berman T., Mizrahi R.i., Dosoretz C.G. (2011). Transparent exopolymer particles (TEP): A critical factor in aquatic biofilm initiation and fouling on filtration membranes. Desalination.

[54] Berman T., Passow U. (2007). Transparent Exopolymer Particles (TEP): An overlooked factor in the process of biofilm formation in aquatic environments. Nat. Preced..

[55] Passow U., Alldredge A.L. (1995). A dye-binding assay for the spectrophotometric measurement of transparent exopolymer particles (TEP). Limnol. Oceanogr..

[56] Wu B., Yi S., Fane A.G. (2011). Microbial community developments and biomass characteristics in membrane bioreactors under different organic loadings. Bioresour. Technol..

[57] Wu B., Yi S., Fane A.G. (2012). Effect of substrate composition (C/N/P ratio) on microbial community and membrane fouling tendency of biomass in membrane bioreactors. Sep. Sci. Technol..

[58] Ahmed Z., Lim B.R., Cho J., Song K.G., Kim K.P., Ahn K.H. (2008). Biological nitrogen and phosphorus removal and changes in microbial community structure in a membrane bioreactor: Effect of different carbon sources. Water Res..

[59] Chen R., LaPara T.M. (2006). Aerobic biological treatment of low-strength synthetic wastewater in membrane-coupled bioreactors: The structure and function of bacterial enrichment cultures as the net growth rate approaches zero. Microb. Ecol..

[60] LaPara T.M., Klatt C.G., Chen R. (2006). Adaptations in bacterial catabolic enzyme activity and community structure in membrane-coupled bioreactors fed simple synthetic wastewater. J. Biotechnol..

[61] Miura Y., Hiraiwa M.N., Ito T., Itonaga T., Watanabe Y., Okabe S. (2007). Bacterial community structures in MBRs treating municipal wastewater: Relationship between community stability and reactor performance. Water Res..

[62] Feng S., Zhang N., Liu H., Du X., Liu Y., Lin H. (2012). The effect of COD/N ratio on process performance and membrane fouling in a submerged bioreactor. Desalination.

[63] Wu B., Yi S., Fane A.G. (2011). Microbial behaviors involved in cake fouling in membrane bioreactors under different solids retention times. Bioresour. Technol..

[64] Zhang J., Chua H.C., Zhou J., Fane A.G. (2006). Effect of sludge retention time on membrane bio-fouling intensity in a submerged membrane bioreactor. Sep. Sci. Technol..

[65] Malamis S., Andreadakis A. (2009). Fractionation of proteins and carbohydrates of extracellular polymeric substances in a membrane bioreactor system. Bioresour. Technol..

[66] Trussell R.S., Merlo R.P., Hermanowicz S.W., Jenkins D. (2006). The effect of organic loading on process performance and membrane fouling in a submerged membrane bioreactor treating municipal wastewater. Water Res..

[67] Gao D.-W., Fu Y., Tao Y., Li X.-X., Xing M., Gao X.-H., Ren N.-Q. (2011). Linking microbial community structure to membrane biofouling associated with varying dissovled oxygen concentrations. Bioresour. Technol..

[68] Ma B.C., Lee Y.N., Park J.S., Lee C.H., Lee S.H., Chang I.S., Ahn T.S. (2006). Correlation between dissolved oxygen concentration, microbial community and membrane permeability in a membrane bioreactor. Process Biochem..

[69] Tocchi C., Federici E., Fidati L., Manzi R., Vincigurerra V., Petruccioli M. (2012). Aerobic treatment of dairy wastewater in an industrial three-reactor plant: Effect of aeration regime on performances and on protozoan and bacterial communities. Water Res..

[70] Jin Y.L., Lee W.N., Lee C.H., Chang I.S., Huang X., Swaminathan T. (2006). Effect of DO concentration on biofilm structure and membrane filterability in submerged membrane bioreactor. Water Res..

[71] Molina-Munoz M., Poyatos J.M., Rodelas B., Pozo C., Manzanera M., Hontoria E., Gonzalez-Lopez J. (2010). Microbial enzymatic activities in a pilot-scale MBR experimental plant under different working conditions. Bioresour. Technol..

[72] Molina-Munoz M., Poyatos J.M., Vilchez R., Hontoria E., Rodelas B., Gonzalez-Lopez J. (2007). Effect of the concentration of suspended solids on the enzymatic activities and biodiversity of a submerged membrane bioreactor for aerobic treatment of domestic wastewater. Appl. Microbiol. Biotechnol..

[73] Calderón K., González-Martínez A., Montero-Puente C., Reboleiro-Rivas P., Poyatos J.M., Juárez-Jiménez B., Martínez-Toledo M.V., Rodelas B. (2012). Bacterial community structure and enzyme activites in a membrane bioreactor (MBR) using pure oxygen as an aerobic source. Bioresour. Technol..

[74] LaPara T.M., Konopka A., Nakatsu C.H., Alleman J.E. (2001). Thermophilic aerobic treatment of a synthetic wastewater in a membrane-coupled bioreactor. J. Indust. Microbiol. Biotechnol..

[75] Molina-Munoz M., Poyatos J.M., Sanchez-Peinado M., Hontoria E., Gonzalez-Lopez J., Rodelas B. (2009). Microbial community structure and dynamics in a pilot-scale submerged membrane bioreactor aerobically treating domestic wastewater under real operation conditions. Sci. Total. Environ..

[76] Simstich B., Beimfohr C., Horn H. (2012). Lab scale experiments using a submerged MBR under thermophilic aerobic conditions for the treatment of paper mill deinking wastewater. Bioresour. Technol..

[77] Miyoshi T., Tsuyuhara T., Ogyu R., Kimura K., Watanabe Y. (2009). Seasonal variation in membrane fouling in membrane bioreactors (MBRs) treating municipal wastewater. Water Res..

[78] van den Brink J.C., Satpradit O.A., van Bentem A., Zwijnenburg A., Temmink H., van loosdrecht M.C. (2011). Effect of temperature shocks on membrane fouling in membrane bioreactors. Water Res..

[79] Drews A., Vocks M., Iversen V., Lesjean B., Kraume M. (2006). Influence of unsteady membrane bioreactor operation on EPS formation and filtration resistance. Desalination.

[80] Wu B., Kitade T., Chong T.H., Uemura T., Fane A.G. (2012). Role of initially formed cake layers on limiting membrane fouling in membrane bioreactors. Bioresour. Technol..

[81] Yogalakshmi K.N., Joseph K. (2010). Effect of transient sodium chloride shock loads on the performance of submerged membrane bioreactor. Bioresour. Technol..

[82] Meng F., Liao B.Q., Liang S., Yang F., Zhang H., Song L. (2010). Morphological visualization, componential characterization and microbiological identification of membrane fouling in membrane bioreactors (MBRs). J. Membr. Sci..

[83] Kniggendorf A.K., Meinhardt-Wollweber M. (2011). Of micropaticles and bacteria identification-(resonance) Raman micro-spectroscopy as a tool for biofilm analysis. Water Res..

[84] Drews A. (2010). Membrane fouling in membrane bioreactors—Characterisation, contradictions, cause and cures. J. Membr. Sci..

[85] Adav S.S., Lin J.C.T., Yang Z., Whiteley C.G., Lee D.J., Peng X.F., Zhang Z.P. (2010). Sterological assessment of extracellular polymeric substances, exo-enzymes, and specific bacterial strains in bioaggregates using fluorescence experiments. Biotechnol. Adv..

[86] Priester J.H., Horst A.M., Van de Werfhorst L.C., Saleta J.L., Mertes L.A., Holden P.A. (2007). Enhanced visualization of microbial biofilms by staining and environmental scanning electron microscopy. J. Microbiol. Methods.

[87] Tian Y., Chen L., Zhang S., Cao C., Zhang S. (2011). Correlating membrane fouling with sludge characteristics in membrane bioreactors: An especial interest in EPS and sludge morphology analysis. Bioresour. Technol..

[88] Tian Y., Chen L., Zhang S., Zhang S. (2011). A sysmetic study of soluble microbial products and their fouling impacts in membrane bioreactors. Chem. Eng. J..

[89] Gönder Z.B., Arayici S., Barlas H. (2011). Advanced treatment of pulp and paper mill wastewater by nanofiltration process: Effects of operating conditions on membrane fouling. Sep. Purif. Technol..

[90] Chen L., Tian Y., Cao C.Q., Zhang J., Li Z.N. (2012). Interaction energy evaluation of soluble microbial products (SMP) on different membrane surfaces: Role of the reconstructed membrane topology. Water Res..

[91] Sweity A., Ying W., Ali-Shtayeh M.S., Yang F., Bick A., Oron G., Herzberg M. (2011). Relation between EPS adherence, viscoelastic properties, and MBR operation: Biofouling study with QCM-D. Water Res..

[92] Chen M.Y., Lee D.J., Yang Z., Peng X.F., Lai J.Y. (2006). Fluorecent staining for study of extracellular polymeric substances in membrane biofouling layers. Environ. Sci. Technol..

[93] Pätzold R., Keuntje M., Theophile K., Muller J., Mielcarek E., Ngezahayo A., Anders-von Ahlften A. (2008). *In situ* mapping of nitrifiers and anammox bacteria in microbial aggregates by means of confocal resonance Raman microscopy. J. Microbiol. Methods.

[94] Haisch C., Niessner R. (2007). Visualisation of transient processes in biofilms by optical coherence tomography. Water Res..

[95] Wagner M., Taherzadeh D., Haisch C., Horn H. (2010). Investigation of the mesoscale structure and volumetric features of biofilms using optical coherence tomography. Biotechnol. Bioeng..

[96] Magic-Knezev A., van der Kooij D. (2004). Optimisation and significance of ATP analysis for measuring active biomass in granular activated carbon filters used in water treatment. Water Res..

[97] Xi C., Wu J. (2010). dATP/ATP, a multifunctional nucleotide, stimulates bacterial cell lysis, extracellular DNA release and biofilm development. PLoS One.

[98] Duan L., Moreno-Andrade I., Huang C.L., Xia S., Hermanowicz S.W. (2009). Effects of short solids retention time on microbial community in a membrane bioreactor. Bioresour. Technol..

[99] Huang L.N., De Wever H., Diels L. (2008). Diverse and distinct bacterial communities induced biofilm fouling in membrane bioreactors operated under different conditions. Environ. Sci. Technol..

[100] Calderon K., Rodelas B., Cabirol N., Gonzalez-Lopez J., Noyola A. (2011). Analysis of microbial communities developed on the fouling layers of a membrane-coupled anaerobic bioreactor applied to wastewater treatment. Bioresour. Technol..

[101] Huber S.A., Balz A., Abert M., Pronk W. (2011). Characterisation of aquatic humic and non-humic matter with size-exclusion chromatography—organic carbon detection—organic nitrogen detection (LC-OCD-OND). Water Res..

[102] Filloux E., Labanowski J., Croue J.P. (2012). Understanding the fouling of UF/MF hollow fibres of biologically treated wastewaters using advanced EfOM characterization and statistical tools. Bioresour. Technol..

[103] Ng T.C., Ng H.Y. (2010). Characterisation of initial fouling in aerobic submerged membrane bioreactors in relation to physico-chemical characteristics under different flux conditions. Water Res..

[104] Kimura K., Naruse T., Watanabe Y. (2009). Changes in characteristics of soluble microbial products in membrane bioreactors associated with different solid retention times: Relation to membrane fouling. Water Res..

[105] Wang Z., Wu Z., Yin X., Tian L. (2008). Membrane fouling in a submerged membrane bioreactor (MBR) under sub-critical flux operation: Membrane foulants and gel layer characterization. J. Membr. Sci..

[106] Smidt E., Parravicini V. (2009). Effect of sewage sludge treatment and additional aerobic post-stabilization revealed by infrared spectroscopy and multivariate data analysis. Bioresour. Technol..

[107] Kimura K., Yamato N., Yamamura H., Watanabe Y. (2005). Membrane fouling in pilot-scale membrane bioreactors (MBRs) treating municipal wastewater. Environ. Sci. Technol..

[108] Jiao Y., Cody G.D., Harding A.K., Wilmes P., Schrenk M., Wheeler K.E., Banfield J.F., Thelen M.P. (2010). Characterization of extracellular polymeric substances from acidophilic microbial biofilms. Appl. Environ. Microbiol..

[109] Lin J.C.T., Lee D.J., Huang C. (2010). Membrane fouling mitigation: Membrane cleaning. Sep. Sci. Technol..

[110] Genkin G., Waite T.D., Fane A.G., Chang S. (2006). The effect of vibration and coagulant addition on the filtration performance of submerged hollow fibre membranes. J. Membr. Sci..

[111] Bilad M.R., Mezohegyi G., Declerck P., Vankelecom I.F. (2012). Novel magnetically induced membrane vibration (MMV) for fouling control in membrane bioreactors. Water Res..

[112] Xiong Y., Liu Y. (2010). Biological control of microbial attachment: A promising alternative for mitigating membrane biofouling. Appl. Microbiol. Biotechnol..

[113] Whitchurch C.B., Tolker-Nielsen T., Ragas P.C., Mattick J.S. (2002). Extracellular DNA required for bacterial biofilm formation. Science.

[114] Camilli A., Bassler B.L. (2006). Bacterial small-molecule signaling pathways. Science.

[115] Hardie K.R., Heurlier K. (2008). Establishing bacterial communities by “word of mouth”: LuxS and autoinducer 2 in biofilm development. Nat. Rev. Microbiol..

[116] Yeon K.M., Cheong W.S., Oh H.S., Lee W.N., Hwang B.K., Lee C.H., Beyenal H., Lewandowski Z. (2009). Quorum sensing: A new biofouling control paradigm in a membrane bioreactor for advanced wastewater treatment. Environ. Sci. Technol..

[117] Xu H., Liu Y. (2011). Control and cleaning of membrane biofouling by energy uncoupling and cellular communication. Environ. Sci. Technol..

[118] Yang X.L., Song H.L., Lu J.L., Fu D.F., Cheng B. (2010). Influence of diatomite addition on membrane fouling and performance in a submerged membrane bioreactor. Bioresour. Technol..

[119] Wu B., An Y., Li Y., Wong F.S. (2009). Effect of adsorption/coagulation on membrane fouling in microfiltration process post-treating anaerobic digestion effluent. Desalination.

[120] Johir M.A.H., Aryal R., Vigneswaran S., Kandasamy J., Grasmick A. (2011). Influence of supporting media in suspension on membrane fouling reduction in submerges membrane bioreactor (SMBR). J. Membr. Sci..

[121] Koseoglu H., Yigit N.O., Civelekoglu G., Harman B.I., Kitis M. (2012). Effects of chemical additives on filtration and rheological characteristics of MBR sludge. Bioresour. Technol..

[122] Wu J., Le-Clech P., Stuetz R.M., Fane A.G., Chen V. (2008). Novel filtration mode for fouling limitation in membrane bioreactors. Water Res..

[123] Teychene B., Guigui C., Cabassud C. (2011). Engineering of an MBR supernatant fouling layer by fine particles addition: A possible way to control cake compressibility. Water Res..

[124] Fane A.G. (1996). Membranes for water production and wastewater reuse. Desalination.

